# Myosin 5 evolution and expression in teleosts

**DOI:** 10.1186/1756-8935-6-S1-P119

**Published:** 2013-04-08

**Authors:** Richard J Nuckels, Dana M Garcia

**Affiliations:** 1 Department of Biology, Texas State University, San Marcos, Texas, USA

## Background

Myosins make up a superfamily of motor proteins best known for their role in actin-based motility. Myosin 5 (*myo5*) is an unconventional myosin that shuttles organelles along cytoplasmic actin tracks. Recently, new roles have been ascribed to myosin 5a and myosin 5b – nuclear roles relating to splicing and transcriptional processing, respectively. Vertebrates have undergone two rounds of whole genome duplication and teleost fishes have undergone an additional round of genome duplication. Consequently *myo5* exists in numerous forms in vertebrates (*myo5a*, *myo5b*, and *myo5c*) with teleosts having duplicates of some of these myosins (*myo5aa*, *myo5ab*, *myo5ba*, and *myo5bb*), but not *myo5c.* A study of the duplicated genes in teleosts may provide insight into the evolutionary history of these genes and their linkage with specific cellular functions, namely transcriptional processing or splicing.

## Materials and methods

RNA and cDNA isolation, RT-PCR and phylogenetic analyses were carried out as described in Nuckels *et al.* 2011 [1].

## Results

An alignment of *myo5a*, *myo5b*, and *myo5c* revealed the conserved regions of the duplicated genes of teleosts. The 5´ myosin head (motor domain) and the 3´ tail (cargo binding) are both highly conserved across taxa and between duplicates, reflecting the importance of these domains in light of 300 million plus years of evolutionary selection. The long neck region of the *myo5* genes is less conserved and contributes to a more robust phylogenetic analysis. Using RT-PCR and qPCR we amplified fragments of *myo5aa*, *myo5ab* and *myo5c* from zebrafish tissues isolated at several developmental time points. This approach revealed differential expression between these duplicates with *myo5aa* being expressed at all developmental time points in whole zebrafish. *myo5aa* is also expressed in whole eyes whereas *myo5ab* seems to be weakly expressed or not expressed at all, depending on the developmental time point.

## Conclusions

Phylogenetic analysis reveals that different regions of the *myo5* genes evolve at different rates. As expected, exons encoding the ATP-binding and motor domains are slowly evolving relative to regions of the gene not involved in a motor or functional domain. Additional studies of expression may reveal subfunctionalization with one duplicate involved in RNA splicing and the other involved in organelle movements, further highlighting the benefit of using a teleost model to study evolutionary consequences of gene duplication.

## References

[B1] Nuckels RJ Forstner MRJ Capalbo-Pitts EL Garcia DM Developmental expression of muscarinic receptors in the eyes of zebrafish Brain Research 2011 1405 85 94 21741623 10.1016/j.brainres.2011.06.016

[B2] de Lanerolle P Serebryannyy L Nuclear actin and myosins: Life without filaments Nature Cell Biology 2011 13 1282 1288 10.1038/ncb2364 22048410

